# A Diacylglycerol Kinase Inhibitor, R-59-022, Blocks Filovirus Internalization in Host Cells

**DOI:** 10.3390/v11030206

**Published:** 2019-03-01

**Authors:** Corina M. Stewart, Stephanie S. Dorion, Marie A. F. Ottenbrite, Nicholas D. LeBlond, Tyler K. T. Smith, Shirley Qiu, Morgan D. Fullerton, Darwyn Kobasa, Marceline Côté

**Affiliations:** 1 Department of Biochemistry, Microbiology and Immunology, University of Ottawa, Ottawa, ON K1H 8M5, Canada; cwark095@uottawa.ca (C.M.S.); sdori068@uottawa.ca (S.S.D.); motte017@uottawa.ca (M.A.F.O.); nlebl062@uottawa.ca (N.D.L.); tsmit121@uottawa.ca (T.K.T.S.); sqiu096@uottawa.ca (S.Q.); morgan.fullerton@uottawa.ca (M.D.F.); 2 Ottawa Institute of Systems Biology, University of Ottawa, Ottawa, ON K1H 8M5, Canada; 3 Centre for Infection, Immunity and Inflammation, University of Ottawa, Ottawa, ON K1H 8M5, Canada; 4 Special Pathogens Program, National Microbiology Laboratory, Public Health Agency of Canada, Winnipeg, MB R3E 3R2, Canada; darwyn.kobasa@canada.ca; 5 Department of Medical Microbiology, University of Manitoba, Winnipeg, MB R3E 0J9, Canada

**Keywords:** Ebola virus, Marburg virus, filoviruses, viral entry, macropinocytosis, diacylglycerol kinase

## Abstract

Filoviruses, such as Ebola virus (EBOV) and Marburg virus, are causative agents of unpredictable outbreaks of severe hemorrhagic fevers in humans and non-human primates. For infection, filoviral particles need to be internalized and delivered to intracellular vesicles containing cathepsin proteases and the viral receptor Niemann-Pick C1. Previous studies have shown that EBOV triggers macropinocytosis of the viral particles in a glycoprotein (GP)-dependent manner, but the molecular events required for filovirus internalization remain mostly unknown. Here we report that the diacylglycerol kinase inhibitor, R-59-022, blocks EBOV GP-mediated entry into Vero cells and bone marrow-derived macrophages. Investigation of the mode of action of the inhibitor revealed that it blocked an early step in entry, more specifically, the internalization of the viral particles via macropinocytosis. Finally, R-59-022 blocked viral entry mediated by a panel of pathogenic filovirus GPs and inhibited growth of replicative Ebola virus. Taken together, our studies suggest that R-59-022 could be used as a tool to investigate macropinocytic uptake of filoviruses and could be a starting point for the development of pan-filoviral therapeutics.

## 1. Introduction

Filoviruses, which include Ebola virus (EBOV) and Marburg virus (MARV), are zoonotic pathogens that can cause unpredictable outbreaks of severe hemorrhagic fevers in humans and non-human primates [1]. The virions are enveloped filamentous particles, of about 80 nm in diameter and an average length of 800-1000 nm, which enclose the negative single-stranded RNA genome of approximately 19,000 nucleotides [2]. The *Filoviridae* family contains three distinct genera: *Ebolavirus* that comprises five viruses (Bundibugyo (BDBV), EBOV, Reston (RESTV), Sudan (SUDV), and Taï Forest (TAFV)), *Marburgvirus*, and *Cuevavirus* [3]. While most outbreaks occurred in Central and West Africa, recent studies have uncovered the high diversity and large geographical distributions of filoviruses including the discovery of new bat-borne filoviruses in China [4]. Although several EBOV vaccines are being developed and the rVSV-EBOV was shown to be efficacious in a phase 3 clinical trial [5,6], these do not protect from infection by all filoviruses. Furthermore, there is currently no FDA-approved antiviral against any of these highly pathogenic viruses.

As enveloped viruses, filoviruses require the fusion of the viral membrane with that of the host cell to deliver its genome into the cell cytoplasm and initiate replication. Membrane fusion is accomplished by the viral glycoprotein (GP) that protrudes from the viral membrane [7]. In the current model of GP triggering for membrane fusion, GP needs to be cleaved by pH-dependent host cathepsin proteases to expose the receptor binding domain, followed by cleaved-GP interaction with the endosome/lysosome resident protein Niemann-Pick C1 (NPC1) [8,9,10,11]. Direct involvement of acidic pH on GP-mediated membrane fusion is still unclear [12,13]. These requirements of a low pH environment and presence host proteins located within intracellular vesicles indicate that a first step in filovirus entry is internalization of the viral particles.

Previous studies have shown that EBOV and potentially all filoviruses use a macropinocytosis or macropinocytosis-like mechanism for internalization [14,15,16,17]. Uptake of EBOV was found to be dependent on Rho GTPases including RhoC, Rac1, and Cdc42 [15,17], p53-activated kinase 1 [14,15], and protein kinase C [15], which are all known to be required for macropinocytosis [18]. While macropinocytosis can be constitutive in some cell types such as macrophages and dendritic cells, it needs to be triggered in others [18]. Interestingly, macropinocytosis was shown to be stimulated by EBOV in a GP-dependent manner as well as by phosphatidylserine molecules present in the viral membrane that can bind to phosphatidylserine receptors expressed by some host cells [14,15,16,19]. The signaling cascades required for filovirus uptake by macropinocytosis remain to be determined.

Macropinocytosis requires large-scale organized movements of the actin cytoskeleton and results in the formation of macropinosomes of diameter varying from 0.2 to 10 μm, which can accommodate the size of filoviral particles [18]. Macropinosome formation requires the generation of membrane ruffles that extend from the cell surface by the assembly of actin filaments [20]. Most ruffles will retract, yet some will bend into cups that will close to form macropinosomes [18]. The lipid composition of the membrane during macropinocytosis - from ruffling, cup formation, to cup closure - is spatio-temporally regulated. For instance, macropinocytosis often requires activation of PI3K for the production of phosphatidylinositol(3,4,5)triphosphate (PtdIns(3,4,5)P_3_) and PtdIns(3,4)P_2_. These lipids can be visualized at the early stages of cup formation [21]. The synthesis of Ins(1,4,5)P_3_ and diacylglycerol (DAG) from PtdIns(4,5)P_2_ by the phospholipase Cγ (PLCγ) are also required. DAG is present in the membrane of the cup at later stages of formation and activates protein kinase Cα [21,22].

DAG kinases (DGKs) are lipid kinases that phosphorylate DAG to generate phosphatidic acid (PA) [23]. In mammals, there are ten isoforms of DGKs. Of these, most of them are localized, at least in part, at the plasma membrane [23]. Recent studies have suggested a role for DGKs in macropinocytosis; DGKζ was required for efficient macropinocytosis following growth factor stimulation [24] and loss of DGKζ expression decreased infection by vaccinia virus, which similarly to EBOV, requires macropinocytosis for viral entry [25]. Whether DGKs are implicated in filovirus entry is currently unknown.

Here we investigated a role for DGK activity in filovirus entry using a specific inhibitor of DGKs, R-59-022. We found that entry of pseudotypes and viral-like particles bearing the EBOV GP, but not those harboring the vesicular stomatitis virus (VSV)-G protein was blocked by R-59-022 in Vero cells. The small-molecule also inhibited EBOV GP-mediated entry in bone marrow-derived macrophages (BMDMs). Further analysis revealed that treatment of cells with R-59-022 led to a drastic inhibition of both virus and high molecular weight dextran internalization. Importantly, R-59-022 blocked entry mediated by multiple filoviral GPs and growth of replication competent EBOV. Our studies suggest that R-59-022 could be used as a tool to dissect the molecular events required for filoviral particle uptake and be a starting point for the development of pan-filoviral inhibitors.

## 2. Materials and Methods

### 2.1. Cell Culture

HEK293T and Vero cells (ATCC) were cultured in Dulbecco’s Modified Eagle Medium (DMEM, Wisent Bioproducts, Saint-Bruno, QC, Canada) and Minimum Essential Medium (MEM, Sigma-Aldrich, St. Louis, MO, USA) respectively, and supplemented with 10% Fetal Bovine Serum (FBS, Sigma-Aldrich), 0.3 mg/mL L-glutamine, 100 U/mL penicillin, and 100 μg/mL streptomycin (Wisent). Cells were maintained at 37 °C in 5% CO_2_ at 100% relative humidity. Bone marrow-derived macrophages (BMDMs) were isolated and differentiated as described previously [26]. Briefly, mice were euthanized, musculature and connective tissue removed from the tibia and femur, and the bones cut at each end. The bones were placed in a tube containing a hole at the bottom, centrifuged, bone marrow cells re-suspended in DMEM supplemented with 10% FBS (Wisent), penicillin and streptomycin (Hyclone, GE Healthcare, Chicago, IL, USA), and were filtered through a 40 µm filter. Cells were differentiated into macrophages in 20% L929-conditioned media and plated for 7 days. On day 8, cells were seeded into 48-well plates and were placed in Roswell Park Memorial Institute medium (RPMI, Wisent), supplemented with 10% FBS (Sigma-Aldrich), 0.3 mg/mL L-glutamine, 100 U/mL penicillin, and 100 µg/mL streptomycin. All animal procedures were approved by the University of Ottawa Animal Care Committee.

### 2.2. Inhibitors and Plasmids

5-(N-ethyl-N-isopropyl)-Amiloride (EIPA, Cayman chemical, Ann Arbor, MI, USA) and R-59-022 (Cayman chemical) were prepared in DMSO, aliquoted, and stored at −80 °C. Ammonium chloride (Sigma-Aldrich) stock solutions of 1.5 M were prepared in water and filtered with 0.22 µm syringe filters.

All plasmids encoding the different virus envelope proteins (EBOV Δmucin GP, BDBV Δmucin GP, SUDV Δmucin GP, or MARV GP and VSV G) described previously [27], the packaging plasmid MLV gag/pol, and the MLV retroviral vector encoding LacZ were kind gifts of Dr. James Cunningham, Brigham and Women’s Hospital. Plasmids encoding EBOV NP and EBOV VP40-β-lactamase were kind gifts of Dr. Lijun Rong, University of Illinois. The mCherry-EBOV VP40 plasmid was a gift from Dr. Judith White (Addgene plasmid # 74421) [28].

### 2.3. Virus Pseudotype and Viral-Like Particle Production

To prepare murine leukemia virus (MLV) pseudotypes, 293T cells were co-transfected with a MLV retroviral vector encoding LacZ, the packaging plasmid MLV gag/pol, and a plasmid encoding the viral glycoprotein of interest (filoviral GP or VSV G). Transfection was performed using the jetPRIME transfection reagent (Polyplus transfection, Illkirch, France) according to the manufacturer’s protocol and at a 1:1:1.15 (LacZ:GagPol:GP) ratio. Supernatants were harvested at 48, 72, and 96 h post-transfection followed by virus concentration by ultracentrifugation (20,000 RPM, 4 °C, 1.5 h, Beckman Coulter Optima XPN-100, SW32Ti rotor) through a 20% (*w*/*v*) sucrose cushion. Concentrated virus was resuspended in PBS, aliquoted, and stored at −80 °C.

To prepare EBOV viral-like particles (VLPs), 293T cells were co-transfected with plasmids encoding the EBOV nucleoprotein (NP), EBOV VP40 fused to β-lactamase (βlam) or mCherry, and the viral glycoprotein of interest (EBOV Δmucin GP, BDBV Δmucin GP, SUDV Δmucin GP, or MARV GP) at a 1:1:1.15 ratio. Transfection and virus concentration was performed as described above.

### 2.4. Virus Entry and Cell Viability Assays

For MLV infection assays, Vero cells were seeded onto white 96-well plates and grown to approximately 60% confluency. Cells were pre-treated for 1 h with inhibitor or vehicle (DMSO, 0.01%) in serum-free MEM containing 5 µg/mL polybrene. Virus was added to pre-treated cells and infection allowed to proceed. Four hours post-infection, the media was replaced with phenol-red free complete DMEM containing 15 mM NH_4_Cl and incubated overnight. Cells were then grown for an additional 48 h in phenol-red free complete DMEM without NH_4_Cl. LacZ+ cells were quantified 72 h post-infection using the Beta-Glo Assay System (Promega, Madison, WI, USA) following the manufacturer’s protocol. Luminescence was read using a Synergy Neo2 Multi-Mode plate reader (BioTek, Winooski, VT, USA).

For VLP entry assays, Vero cells or BMDMs were seeded onto clear 48-well plates and grown to approximately 90% confluency. Cells were pre-treated for 1 h with inhibitor or vehicle in serum-free MEM (for Vero cells) or serum-free RPMI (for BMDMs) followed by the addition of VLPs. Three hours post-infection, cells were stained for 2 h at room temperature. Staining solution was prepared using the CCF2-AM kit (ThermoFisher, Waltham, MA, USA) according to the manufacturer’s protocol and supplemented with 15 mM NH_4_Cl and 250 µM probenecid (Sigma-Aldrich). After staining, cells were washed, trypsinized, and prepared for analysis by flow cytometry (FACSCelesta, BD Biosciences, Franklin Lakes, NJ, USA). Infection was quantified using the FlowJo software by assessing the percentage of cells with cleaved CCF2 (shift from 530 nm to 460 nm emission) compared to uninfected samples. Cell viability/metabolic activity was also measured 4 h post-inhibitor incubation using the CellTitre-Glo assay system (Promega) according to the manufacturer’s protocol. Luminescence was read using a Synergy Neo2 Multi-Mode plate reader (BioTek).

### 2.5. Time of Addition Assay

Vero cells were seeded onto clear 48-well plates and grown to approximately 90% confluency. Media was changed to cold serum-free MEM and cells incubated at 4 °C for 15 min. βlam VLPs were added on ice and attached to the surface of the cells by centrifugation at 300× *g* for 30 min at 4 °C. Inhibitors or NH_4_Cl were added at the time of infection (0 h) or various time points post-infection (15 min, 30 min, 60 min, 120 min, 150 min). For the DMSO control, vehicle was added at the 0 h time point. The infection was allowed to proceed and samples analyzed by flow cytometry as described above.

### 2.6. Virus Attachment Assay

Vero cells were detached using 0.5 mM EDTA in phosphate buffered saline (PBS), counted, and 100,000 cells were aliquoted into tubes. Cells were spun down, re-suspended in 2% FBS in PBS containing inhibitor or vehicle, and incubated at 37 °C for 30 min. Cells were then incubated at 4 °C for 15 min, followed by addition of mCherry VLPs and incubation at 4 °C for 1 h. Cells were washed twice with cold PBS, re-suspended in cold 2% FBS in PBS, and mCherry fluorescence analyzed by flow cytometry (Celesta, BD Biosciences). In the trypsinized condition, 0.05% trypsin was added after the cold PBS washes and incubated for 5 min at room temperature prior to centrifugation and resuspension in cold 2% FBS in PBS.

### 2.7. Virus Internalization Assay

Vero cells or BMDMs were seeded onto clear 48-well plates and grown to approximately 90% confluency. Cells were pre-treated for 30 min with inhibitor or vehicle in serum-free MEM (for Vero cells) or serum-free RPMI (for BMDMs). Cells were incubated at 4 °C for 15 min and mCherry VLPs added and attached to the surface of the cells by centrifugation at 300× *g* for 30 min at 4 °C. Unbound VLPs were washed with cold PBS followed by the addition of media containing pre-warmed inhibitor or vehicle and cells were incubated at 37 °C for 1 h. For the 4 °C control, cold media was added after the PBS wash instead of pre-warmed media and the incubation was maintained at 4 °C for the duration of the assay. After 1 h, the cells were again washed with cold PBS and incubated at 4 °C with 0.5% trypsin for 30 min. Samples were prepared and mCherry fluorescence analyzed by flow cytometry (Celesta, BD Biosciences).

### 2.8. Dextran Internalization Assay

Vero cells were seeded onto coverslips and grown to approximately 60% confluency. Cells were pre-treated for 1 h with inhibitor or vehicle in serum-free MEM, followed by a 30 min incubation with Dextran-Fluorescein (10,000 MW, Anionic, 1 mg/mL, ThermoFisher) and CellTracker Blue CMAC Dye (ThermoFisher). Cells were washed, fixed, and imaged by confocal microscopy (LSM800 AxioObserver Z1, Zeiss, Oberkochen, Germany). Image analysis was performed using Imaris image analysis software (Bitplane, Zurich, Switzerland) whereby dextran containing vesicles were modeled as spots and counted for each cell.

### 2.9. Live-Cell Imaging of VLPs and Actin

Vero cells were seeded to approximately 70% confluency and transfected with a plasmid encoding GFP-Utrophin, a gift from William Bement (Addgene plasmid # 26737) [29], using jetPRIME transfection reagent (Polyplus transfection) according to the manufacturer’s protocol. Twenty-four hours post-transfection, cells were re-seeded onto chamber slides (Lab-Tek, Scotts Valley, California, USA) to about 60% confluency. The next day, cells were pre-incubated with inhibitor or vehicle and mCherry EBOV VLPs were added. Live-cell imaging was performed immediately after addition of VLPs and proceeded for no longer than 30 min. The live imaging was performed on a Zeiss LSM 880 AxioObserver Z1 confocal microscope using AiryScan FAST mode on a single z plane. The environmental control chamber was set to 37 °C, 5% CO_2_ and a 63x/1.4NA oil objective was used. Image analysis was performed using Imaris software (Bitplane) by tracking modeled VLPs as spots over time using the Brownian motion tracking algorithm.

### 2.10. Replication-Competent Virus Growth Assay

VeroE6 cells were seeded in clear bottom, black well tissue culture plates (Corning, Kennebunk, ME, USA) to be 80% confluent at the time of infection. The cells were treated with different concentrations of R-59-022 and infected with EBOV (strain Mayinga), expressing enhanced-GFP, at a multiplicity of infection of 0.1. Virus growth was assessed by measurement of GFP at different time-points using a BioTek Synergy/HTX plate reader with excitation at 485 nm and emission at 516 nm. Experiments with replication-competent EBOV were performed in the Biosafety Level 4 facility at the National Microbiology Laboratory at the Public Health Agency of Canada.

### 2.11. Statistical Analysis

**Figure 1:**
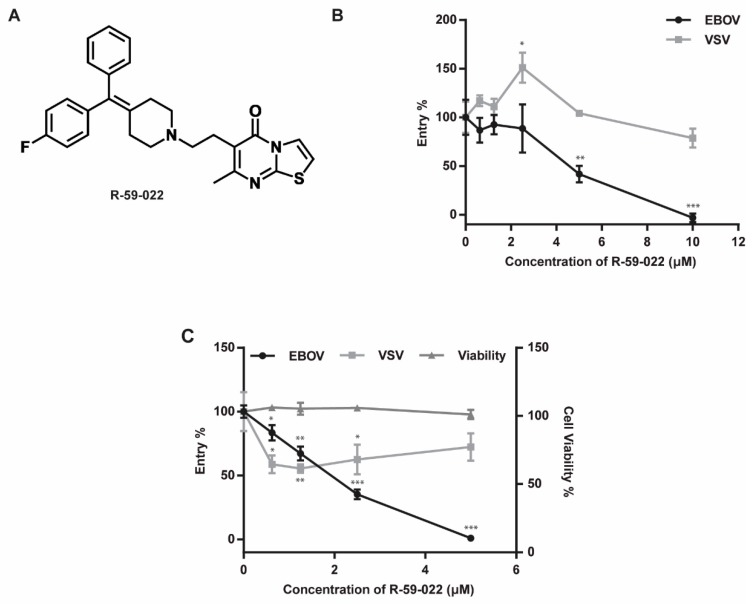
R-59-022 blocks EBOV GP-mediated entry in Vero cells. (**A**) Chemical structure of R-59-022; (**B**,**C**) Measurement of GP-mediated entry in Vero cells. Vero cells were pre-treated for 1 h with increasing concentrations of R-59-022 or vehicle and exposed to (**B**) MLV pseudotypes encoding LacZ or (**C**) βlam VLPs harbouring the indicated viral glycoproteins in the presence of the drug for 4 and 3 h respectively. Infection was measured by (**B**) quantifying β-galactosidase activity using a luminescent substrate or (**C**) determining the percentage of cells with cytoplasmic βlam by CCF2-AM staining and flow cytometry analysis. Data are expressed as percentages relative to vehicle-treated cells; (**C**) Metabolic activity of the inhibitor-treated cells was measured in parallel and values were normalized to vehicle-treated cells. Data are representative of 3 independent experiments. * *p* < 0.05, ** *p* < 0.01, *** *p* < 0.001.

**Figure 2:**
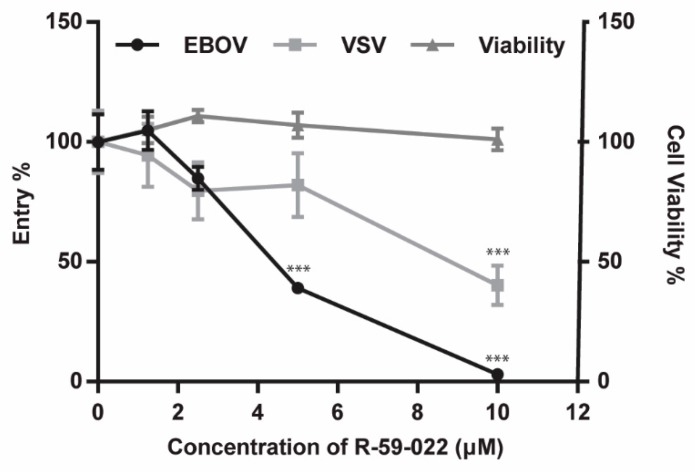
R-59-022 inhibits EBOV GP-mediated entry in bone marrow-derived macrophages. BMDMs were pre-treated for 1 hour with increasing concentrations of R-59-022 or vehicle and exposed to βlam VLPs harbouring EBOV GP or VSV G. Infection was measured by determining the percentage of cells with cytoplasmic βlam by CCF2-AM staining and flow cytometry analysis. Data are expressed as percentages relative to vehicle-treated cells. Metabolic activity was measured in parallel and was also normalized to vehicle-treated cells. Data are representative of 3 independent experiments. * *p* < 0.05, ** *p* < 0.01, *** *p* < 0.001.

**Figure 3:**
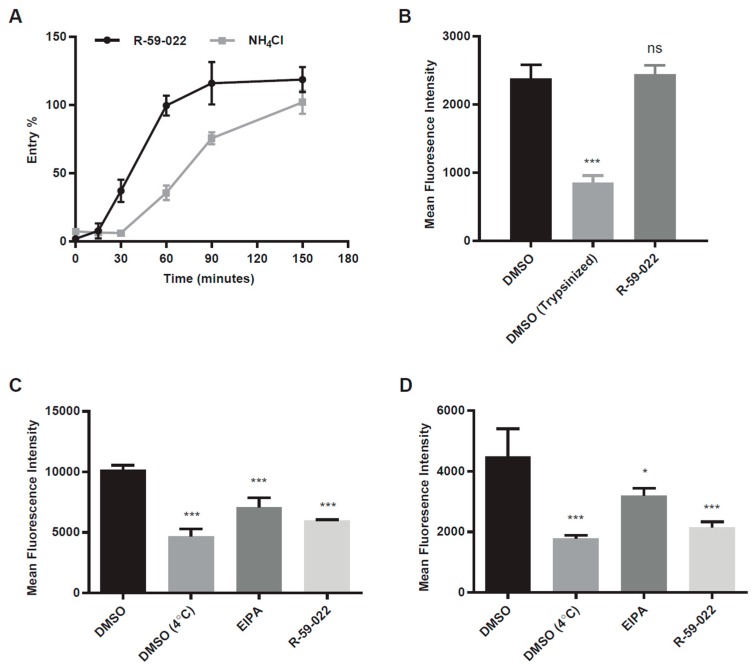
R-59-022 blocks internalization of EBOV VLPs into the host cell. (**A**) Time of addition assay in Vero cells with βlam VLPs harboring EBOV GP. Cells were infected at *t* = 0 and R-59-022 (5 µM) or NH_4_Cl (15 mM) were added at indicated time points post-infection. Viral entry was measured by determining the percentage of cells with cleaved CCF2 compared to vehicle; (**B**) Attachment assay in Vero cells using mCherry VLPs harbouring EBOV GP. Vero cells were pre-treated with 5 µM R-59-022 or vehicle followed by a 1-hour incubation at 4 °C with EBOV VLPs. One set of DMSO samples was trypsinized to remove bound VLPs. Fluorescence of mCherry VLPs was measured by flow cytometry and mean fluorescence intensity (MFI) for each sample determined using the FlowJo software; (**C**,**D**) Internalization assay in (**C**) Vero cells and (**D**) BMDMs using mCherry EBOV VLPs. Cells were pre-treated with 5 µM R-59-022, 30 µM EIPA, or vehicle followed by addition of VLPs and spinoculation at 4 °C. Cells were washed with cold PBS and pre-warmed media containing inhibitor or vehicle was added. Cells with attached VLPs were incubated at 37 °C for 1 h to allow for internalization and were then moved to 4 °C for 15 min. Cells were then trypsinized at 4 °C for 30 min to remove non-internalized VLPs. Fluorescence of internalized mCherry VLPs was measured by flow cytometry and mean fluorescence intensity determined using the FlowJo software. Data are representative of 3 independent experiments. * *p* < 0.05, ** *p* < 0.01, *** *p* < 0.001, ns: not significant.

**Figure 4:**
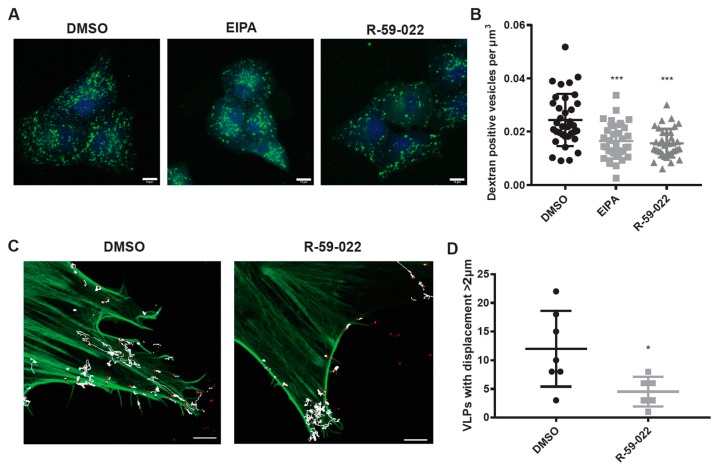
R-59-022 inhibits macropinocytosis in Vero cells. (**A**) Vero cells were pre-treated with R-59-022 (5 µM), EIPA (30 µM), or vehicle for 30 min followed by addition of high molecular weight fluorescein dextran and incubation at 37 °C for another 30 min. Cells were stained with Cell Tracker Blue CMAC fluorescent dye, which was added concomitantly with the fluorescent dextran. Cells were fixed and imaged with a LSM 800 confocal microscope (Zeiss). Images are displayed as maximum intensity z-projections, bar = 10 µm; (**B**) Number of dextran-positive puncta per cell volume (µm^3^) was determined using the Imaris software (Bitplane). (**C**) GFP-Utrophin transfected Vero cells were pre-treated with R-59-022 (5 µM) or vehicle for at least 30 min. The cells were then placed in an environmental chamber (37 °C, 5% CO_2_) and mCherry EBOV VLPs added. Immediately after VLP addition, cells were imaged with a LSM 880 confocal microscopy (Zeiss) using AiryScan FAST on a single z plane. Images were analyzed using tracking algorithms on Imaris software (Bitplane). Each track represents the movement of VLPs over time. Bar = 5 µm; (**D**) Track length and displacement were measured (per VLP) and the number of VLPs with a displacement greater than 2 µM were counted per time lapse image. Data are representative of 3 independent experiments. * *p* < 0.05, *p* < 0.01, *** *p* < 0.001.

Statistical analysis was performed using Prism (GraphPad Software, San Diego, CA, USA). T-tests were performed for figures 1,2 and 4D and one-way ANOVA was used for figure 3B–D. Data in all figures are means ± standard deviation.

## 3. Results

### 3.1. R-59-022 Efficiently Blocks EBOV GP-Mediated Entry into Vero Cells

To investigate a potential role for DAG kinases in filovirus entry, we first tested the effect of a commercially available DAG kinase inhibitor R-59-022 on EBOV GP-mediated entry (Figure 1A). Because previous studies have shown that the mucin region of GP is not required for uptake via macropinocytosis or for cathepsin/NPC1 dependence, we used a mucin-deleted version of EBOV GP, which allows higher viral particle yields [8,9,16]. Vero cells were treated with increasing concentrations of R-59-022 and exposed for 4 hours to MLV pseudotypes encoding LacZ and bearing EBOV GP or VSV-G in the presence of the drug. Media was then replaced with media containing ammonium chloride to stop further viral entry. Successful entry and provirus integration were then assessed by measurement of β-galactosidase activity using a luminescent substrate. We found that the inhibitor blocked entry of EBOV pseudotypes in a concentration-dependent manner (IC50: ~5µM, Figure 1B), but had no significant inhibitory effect on VSV pseudotypes even at the highest concentration tested (Figure 1B). Instead, a slight increase in VSV pseudotype infection was observed at low R-59-022 concentrations. These results strongly suggested that R-59-022 blocked an entry step specific to EBOV GP.

To directly test for a potential role of R-59-022 in blocking viral entry, we used viral-like particles (VLPs) produced by co-transfection of EBOV NP and VP40 in cells. Expression of these viral proteins leads to the production of filoviral-like particles of similar morphology and size to those of native filoviral particles. To allow and measure virus-cell fusion of these particles into target cells, VLPs were produced by co-transfection of plasmids encoding EBOV NP, an EBOV VP40 construct fused with β-lactamase (βlam), and EBOV GP or VSV-G. The entry of these βlam VLPs leads to the delivery of VP40-βlam into the target cell cytoplasm which can be detected by loading cells with the βlam FRET substrate CCF2-AM. Vero cells were treated with increasing concentrations of R-59-022 and exposed to VLPs bearing EBOV GP or VSV G in the presence of the drug for 3 h. Delivery of VP40-βlam into the target cell cytoplasm was then assessed by labeling with CCF2-AM and analyzed by flow cytometry. Using this assay, we observed a R-59-022 dose-dependent decrease in entry by the VLPs harboring the EBOV GP (IC50: ~2 µM, Figure 1C). Interestingly, unlike the results of the experiments using MLV pseudotypes, a slight decrease in VSV G-mediated entry could also be observed. However, the decrease in entry of the VLPs bearing VSV-G was not dose-dependent (Figure 1C). This decrease can potentially be attributed to the large size some of the filoviral-like particles can exhibit, especially when compared to the size of MLV particles. Importantly, the inhibitor treatment did not have a detectable effect on the viability of Vero cells even at the highest concentration (Figure 1C). These results demonstrate that R-59-022 is an inhibitor of EBOV-GP mediated entry.

### 3.2. EBOV GP-Mediated Entry in Bone Marrow-Derived Macrophages is Inhibited by R-59-022

One of the primary targets of EBOV *in vivo* is macrophages. Therefore, we next sought to test if R-59-022 could also block EBOV GP-mediated entry in bone marrow-derived macrophages (BMDMs). Because BMDMs are terminally differentiated and do not proliferate, MLV pseudotypes could not be used for these experiments as MLV requires actively dividing cells for integration. Thus, we directly assessed EBOV GP-mediated entry in the presence of the inhibitor using the βlam VLP bearing EBOV GP or VSV-G. BMDMs were treated with increasing concentrations of R-59-022 and exposed to the βlam VLPs in the presence of the drug for 3 hours before staining and analysis by flow cytometry. Similarly to the results obtained with Vero cells, we found that entry of EBOV VLPs was significantly inhibited by R-59-022 in a dose-dependent manner (Figure 2). A reduction in the entry of the VSV-G VLPs could also be observed, albeit at a lesser extent when compared to the EBOV GP VLPs. Unlike the decrease obtained in the Vero cells, the slight decrease of VSV-G VLPs in the BMDMs was dose-dependent suggesting that VSV-G entry in those cells is in part sensitive to R-59-022 (Figure 2). Again, the drug treatment had no noticeable effect on cell viability when using Cell-Titer Glo to measure metabolic activity of the cells (Figure 2). Taken together, our data suggest that R-59-022 can inhibit EBOV GP-mediated entry in multiple cell types.

### 3.3. R-59-022 Interferes with Virus Internalization

EBOV entry involves multiple steps including attachment to the target cell, internalization by a macropinocytosis or macropinocytosis-like mechanism, trafficking, cleavage of GP by acid-dependent cathepsin proteases, and GP interaction with NPC1 [30]. To identify the step at which R-59-022 inhibits EBOV GP-mediated entry, we first performed kinetics experiments to determine if the drug acted at an early or late step of viral entry. Vero cells were exposed to βlam VLPs bearing EBOV GP and R-59-022 was added at different time-points during infection. Ammonium chloride (NH_4_Cl) was used as a control as this lysosomotropic agent neutralizes the pH of the endosomes and lysosomes and therefore inhibits cathepsin activity and potentially downstream membrane fusion events. Using this assay, we found that the inhibitory activity of R-59-022 was lost at earlier time points when compared to that of NH_4_Cl (Figure 3A). This result suggested that R-59-022 blocked an early step of EBOV GP-mediated viral entry.

The first steps in EBOV entry are virus attachment and internalization. To assess the ability of R-59-022 to interfere with virus attachment, we produced fluorescent VLPs, by the co-expression of a VP40 construct fused with mCherry. Vero cells were incubated with mCherry VLPs in the presence or absence of the inhibitor at 4 °C to prevent internalization. Cells were washed to remove unbound viral particles, and the attached VLPs were measured using flow cytometry. Using the IC90 concentration of R-59-022, no significant difference in the extent of virus attachment was observed in the presence drug when compared to vehicle-treated cells (Figure 3B). To determine if R-59-022 blocked internalization, cells were incubated with mCherry VLPs at 4 °C, unbound particles were removed, and cells with mCherry VLPs were incubated at 37 °C to allow for internalization. For these experiments, 5-(N-Ethyl-N-isopropyl)amiloride (EIPA), a known inhibitor of macropinocytosis, was used as a control. We found that R-59-022 significantly inhibited VLP internalization at levels similar to that of EIPA treatment (Figure 3C). To confirm that the inhibitor has the same mode of action in different cell types, we also tested internalization of VLPs in the presence of R-59-022 in BMDMs. Again, internalization of VLPs was significantly blocked by the inhibitor (Figure 3D). Interestingly, R-59-022 was even more potent than EIPA at inhibiting VLP internalization in these cells. Taken together, our results indicate that R-59-022 interferes with EBOV VLP internalization.

### 3.4. R-59-022 Blocks Macropinocytosis in Vero Cells

Since VLPs with EBOV GP are internalized via macropinocytosis or a macropinocytosis-like mechanism, we hypothesized that R-59-022 inhibits macropinocytosis. To test this, we incubated Vero cells with high molecular weight fluorescently labeled dextran, known to enter cells via macropinocytosis, in the presence or absence of R-59-022 or EIPA. As expected, treatment with R-59-022 and EIPA reduced the number of dextran positive vesicles in cells (Figure 4A,B). These results indicate that R-59-022 inhibits macropinocytosis.

Our results suggest that R-59-022 blocks macropinocytic uptake of viral particles in cells. In an attempt to determine the fate of the VLPs in the presence of R-59-022, we used live-cell imaging to visualize the behavior of fluorescent VLPs. In addition, we sought to monitor actin filaments using GFP-utrophin as actin rearrangements are required for macropinocytosis. GFP-Utrophin transfected Vero cells were treated with vehicle or R-59-022 and mCherry VLPs bearing EBOV GP were added immediately prior to imaging. Interestingly, we observed significantly less movements of VLPs on/in the cell in the presence of R-59-022 treated cells compared to vehicle alone (Figure 4C,D, Videos S1–S4). While VLPs were able to attach to the surface of the R-59-022 treated cell, these VLPs appeared to be ‘stuck’ and fewer actin cups were observed (Videos S1–S4). In sum, we observed a defect in macropinocytic uptake of high molecular weight dextran and reduced movements of VLPs in the presence of R-59-022.

### 3.5. R-59-022 Blocks Viral Entry of Pathogenic Filoviruses

**Figure 5:**
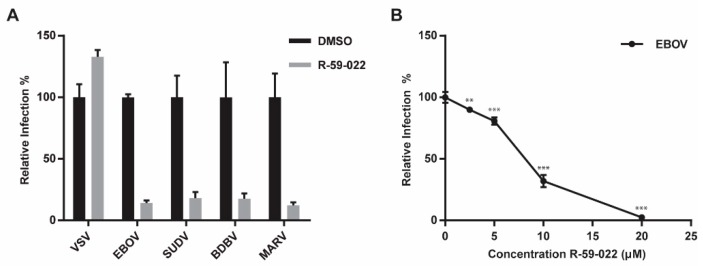
R-59-022 blocks entry of pathogenic filoviruses and growth of replication-competent EBOV (**A**) Infection of Vero cells with βlam VLPs harbouring the GPs of EBOV, SUDV, BDBV, MARV, or VSV G in the presence of 5 µM R-59-022 or vehicle. Infection was measured using flow cytometry by determining the percentage of cells with cleaved CCF2. Data are expressed as percentages relative to vehicle-treated cells; (**B**) Infection of Vero cells with replication-competent EBOV expressing GFP at increasing concentrations of R-59-022 or vehicle. Infection was measured by GFP fluorescence 3 days post-infection and normalized to vehicle treated cells. Data is representative of 3 independent experiments. * *p* < 0.05, ** *p* < 0.01, *** *p* < 0.001.

Our data suggest that R-59-022 blocks macropinocytic uptake of EBOV VLPs. Given the large size of all filoviruses and the dependence on triggering factors such as NPC1 located in intracellular vesicles, it is expected that all filoviruses will require uptake by macropinocytosis or a macropinocytosis-like mechanism. Therefore, the prediction is that entry of all filoviruses will be blocked by R-59-022. To test this, we produced βlam VLPs harboring the GP of a panel of pathogenic filoviruses; EBOV, BDBV, SUDV, and MARV. Vero cells were exposed to the different VLPs in the presence of R-59-022 and entry efficiency was measured by CCF2-AM staining and flow cytometry analysis. As expected, entry by the VLPs harboring the filovirus GPs were potently blocked by R-59-022, while entry mediated by VSV G was not affected at the concentration used (Figure 5A). These data indicate that R-59-022 blocks a step used by all pathogenic filoviruses, presumably macropinocytosis, and could therefore be a pan-filovirus inhibitor.

### 3.6. EBOV Growth is Inhibited by R-59-022

As a preliminary assessment of R-59-022 potential as an anti-filovirus therapeutic, we tested the inhibitor for activity against replication-competent EBOV. Vero cells were treated with different concentrations of R-59-022 and infected with EBOV Mayinga expressing GFP. Virus growth was assessed by measurement of GFP at different time-points. Using this assay, we found that R-59-022 could indeed block EBOV growth, although it required higher concentrations then those used for single round infections (Figure 5B). Taken together, our results suggest that R-59-022 can inhibit native EBOV and potentially other filoviruses.

## 4. Discussion

The entry mechanism of filoviruses involves a complex series of events that culminates with the fusion of the viral membrane with the endosomal membrane of the cell. Viral particles need to be internalized via macropinocytosis and trafficked to endosomal compartments containing cathepsin proteases and the viral receptor NPC1. While the molecular details of how each step is regulated remain to be investigated, we report here the discovery of R-59-022, a small-molecule inhibitor of filovirus entry. Analysis of the inhibitory mechanism of R-59-022 revealed that it prevented the macropinocytic uptake of filoviral particles, inhibited entry mediated by multiple filovirus GPs, and blocked replicative EBOV growth.

Several lines of evidence indicate that R-59-022 is an inhibitor of macropinocytosis. First, investigation of the kinetics of action of the inhibitor revealed that it specifically interfered with an early step in entry (Figure 3A). Second, further analysis of attachment and internalization of filoviral particles indicated that virus uptake was prevented in the presence of the drug (Figure 3C,D) and live-imaging of viral particles in R-59-022 treated cells showed that viruses remained mostly static at the surface of the cells (Figure 4C,D). Third, internalization of high molecular weight dextran, which is known to be primarily mediated by macropinocytosis [14], was also inhibited in the presence of the drug (Figure 4A,B). Finally, while R-59-022 blocked entry mediated by all filovirus GPs tested (Figure 5A), it had no striking effect on VSV G-mediated entry which was shown to be internalized via clathrin-dependent endocytosis [31]. Interestingly, low concentrations of R-59-022 caused a small increase in VSV MLV pseudotype infection (Figure 1B). Whether low concentrations of R-59-022 can increase clathrin-mediated endocytosis remains to be determined. In contrast, we did observe a slight reduction in the entry of filoviral-like particles harbouring VSV G (Figure 1C). Given that these particles will exhibit the characteristic heterogenous filamentous morphology of EBOV, which can reach 1000 nm in length, it is expected that vesicles formed by clathrin-coated pits will be unable to accommodate the viral particles with larger sizes. Indeed, the biggest particles should only be able to enter cells via macropinocytosis or phagocytosis regardless of the viral glycoprotein expressed at their surface. Consequently, the slight decrease in VSV G-mediated entry of filoviral-like particles can be explained by a block of internalization of a small fraction of the heterogenous particle population that comprises particles that are too large and require uptake by macropinocytosis. Therefore, our studies identify R-59-022 as a macropinocytosis inhibitor.

R-59-022 adds to the known repertoire of macropinocytosis-targeting EBOV inhibitors. Commonly used macropinocytosis inhibitors for viral entry include EIPA and Latrunculin A (Lat A). Each, however, have their own limitations and drawbacks. For example, EIPA, an analogue of the prototypical amiloride, is an inhibitor of Na+/H+ exchange that must be used at high concentrations of at least 25 µM, increasing off-target effects and reducing specificity. In addition, a study using sensitive pH nanosensors has also showed that amiloride treatment leads to change in endosomal pH [32]. With regards to Lat A, being a natural product that sequesters monomeric actin, it interferes with cell morphology and cytoskeletal homeostasis in addition to blocking macropinocytosis [33]. Although more characterization of the mode of action R-59-022 is required, R-59-022 has the potential to be a useful tool for the study of macropinocytosis.

Our results show that R-59-022 inhibited the uptake of filoviral particles in both Vero cells and BMDMs. Interestingly, BMDMs can potentially also internalize the VLPs by phagocytosis which is a process that closely resembles macropinocytosis [18]. Analysis of EBOV GP-mediated entry of VLPs in the presence of R-59-022 revealed that the drug inhibited entry completely at 10 μM. While more work needs to be done, these results indicate that R-59-022 can inhibit macropinocytosis in multiple cell types and can also potentially interfere with phagocytosis. In addition, since R-59-022 blocked high molecular weight dextran uptake, this suggest that R-59-022 could also inhibit other viruses in addition to large molecules and complexes. Future studies will be needed to determine if R-59-022 can also inhibit other intracellular pathogens that utilize macropinocytosis and/or phagocytosis to enter the host cell.

Previous studies have demonstrated that EBOV triggers signaling cascades during viral entry. Cellular signaling proteins, such as Akt, PI3K, and the PIKfyve/ArPIKfyve/Sac3 complex, were shown to be activated during EBOV entry and involved in the intracellular trafficking and delivery of viral particles to vesicles containing NPC1 [34,35]. However, how EBOV regulates and/or induces macropinocytic uptake remain to be fully elucidated. Previous studies have shown that AMP-activated protein kinase is required for uptake of EBOV in multiple cell types [36]. More recently, the exchange protein directly activated by cAMP was also shown to be important for EBOV uptake in endothelial cells [37]. R-59-022 targets DGKs, which are relatively uncharacterized regulators of macropinocytosis, and represents to our knowledge the first evidence that EBOV could require these host enzymes. Future studies will be focused on the validation of the antiviral target of R-59-022 and investigation into a possible role for the ten different DGK isozymes in filovirus entry.

In this study, we have identified a small-molecule that blocks filovirus infection. To date, there are no FDA-approved antiviral therapies for EBOV or other filoviruses, highlighting the need for the identification of new small- molecule inhibitors of infection. We have found that R-59-022 is an inhibitor of filovirus internalization, therefore representing a potential starting point for the development of a pan-filovirus therapy.

## Supplementary Materials

The following are available online at https://www.mdpi.com/1999-4915/11/3/206/s1, Video S1: DMSO A, Video S2: DMSO B, Video S3: R-59-022 A, Video S4: R-59-022 B.
